# The Fundamental Role of Bicarbonate Transporters and Associated Carbonic Anhydrase Enzymes in Maintaining Ion and pH Homeostasis in Non-Secretory Organs

**DOI:** 10.3390/ijms21010339

**Published:** 2020-01-04

**Authors:** Dongun Lee, Jeong Hee Hong

**Affiliations:** Department of Physiology, Lee Gil Ya Cancer and Diabetes Institute, College of Medicine, Gachon University, Incheon 21999, Korea; sppotato1@gmail.com

**Keywords:** bicarbonate, ion transporters, carbonic anhydrase, intracellular pH, maturation

## Abstract

The bicarbonate ion has a fundamental role in vital systems. Impaired bicarbonate transport leads to various diseases, including immune disorders, cystic fibrosis, tumorigenesis, kidney diseases, brain dysfunction, tooth fracture, ischemic reperfusion injury, hypertension, impaired reproductive system, and systemic acidosis. Carbonic anhydrases are involved in the mechanism of bicarbonate movement and consist of complex of bicarbonate transport systems including bicarbonate transporters. This review focused on the convergent regulation of ion homeostasis through various ion transporters including bicarbonate transporters, their regulatory enzymes, such as carbonic anhydrases, pH regulatory role, and the expression pattern of ion transporters in non-secretory systems throughout the body. Understanding the correlation between these systems will be helpful in order to obtain new insights and design potential therapeutic strategies for the treatment of pH-related disorders. In this review, we have discussed the broad prospects and challenges that remain in elucidation of bicarbonate-transport-related biological and developmental systems.

## 1. Convergent Regulation of Ion Homeostasis

Ion homeostasis is an important process involved in various organ functions, including modulation of sensitivity to blood pressure, immune cell differentiation, fluid secretion, and fertilization of reproductive cells such as sperm and eggs. Ions utilize several paths to enter the cytosolic milieu, by accompanying other cations and anions through the involvement of cotransporters or energy-consuming channels. In this section, we have focused on the convergent regulation of various ion transporters and channels in various physiological and pathophysiological milieus.

Na^+^ is an essential element for all living organisms and a crucial ion for the modulation of osmotic pressure. Regulation of Na^+^ concentration is modulated by various ion transporters, including Na^+^-K^+^-Cl^−^ cotransporter 1 (NKCC1), electrogenic-type Na^+^-HCO_3_^−^ cotransporter (NBCe), and sodium–potassium ATPase (Na^+^-K^+^-ATPase). Coregulation of these transporters and a pump revealed that cytosolic Na^+^ level is strongly dependent on the extracellular K^+^ and HCO_3_^−^ levels in cortical astrocytes, which are involved in pH regulation and volume regulation [1]. The HCO_3_^−^-dependent channels and transporters include cystic fibrosis transmembrane conductance regulator channel (CFTR) [2,3], NBC [4,5], Cl^−^/HCO_3_^−^ exchangers (CBE) [6,7], and Ca^2+^-activated Cl^−^ channel TMEM16A (also known as Anoctamin-1) [8,9]. The NBC and the epithelial sodium channel (ENaC) are essential in the modulation of CFTR-dependent membrane potential and the cAMP/protein kinase A (PKA) signaling pathway in sperm capacitation [10]. The CFTR exists in epithelial cells and also exhibits critical functions in lung homeostasis related to maintain the ion gradients. The mutation of CFTR, ΔF508 CFTR, causes chronic inflammatory responses and. eventually, cystic fibrosis (CF) [11]. The function of CFTR reciprocally regulates ENaC activity. In CF patients, ΔF508 CFTR and highly activated ENaC coexist in lung epithelia. Overexpressed wild-type CFTR effectively suppresses ENaC activity, whereas ΔF508 CFTR elevated ENaC activity in a CF airway epithelial model, suggesting that CFTR is functionally involved in regulation of inflammatory responses in the lungs [11].

The regulation of intracellular pH (pH_i_) is mediated by HCO_3_^−^ transporters coupled with Na^+^, including NBCe, electroneutral-type NBC (NBCn), and Na^+^-driven CBE (NDCBE) [12]. A splice variant of NBCe, NBCe2, is associated with salt sensitivity in the renal proximal tubule system. Although NBCe2 modulates renal HCO_3_^−^ transport independently of hypertension, NBCe2 activity is hyperresponsive to intracellular Na^+^ concentration [13]. The Na^+^-H^+^ exchanger (NHE) and CBE activity were also higher in homozygous variants compared to wild-type variants of NBCe2 in the renal proximal tubule cells [14]. Involvement of NHE in the enhanced NBC system can be implicated in the regulation of steady-state pH_i_. Although this evidence was gathered in vascular smooth muscle cells, elevated steady-state pH_i_ and enhanced pH recovery from acidosis are mediated by NBCn1. Association of NHE activity maintains a steady-state pH_i_ [15]. NDCBE, such as that coded by gene *SLC4A8*, transports extracellular Na^+^ and HCO_3_^−^ into cells in exchange for intracellular Cl^−^ in brain and testis [12]. In contrast, one of the CBEs, anion exchanger 4 (AE4), promotes Cl^−^ influx to exchange K^+^ (or Na^+^) for HCO_3_^−^ in secretory cells [16]. A recent study demonstrated that Cl^−^/HCO_3_^−^ exchange of AE4 under Cl^−^-free conditions mediates Na^+^-HCO_3_^−^ cotransport without changes in membrane potential [16]. In addition, the transport of Na^+^, Cl^−^, and HCO_3_^−^ occurs in the presence of K^+^, along with Cs^+^, Li^+^, and Rb^+^ [16]. AE3, the functions of which are still unknown, has been implicated in acid–base homeostasis in the kidney [17]. However, Kampik et al. showed that expression of transient receptor potential melastatin (TRPM) 6 channel increased in the kidney of AE3-deficient mice [17]. Another CBE, AE1, harbors a binding site of the Na^+^-K^+^-ATPase β1 subunit, especially in the human kidney [18]. Interaction between AE1 and the Na^+^-K^+^-ATPase (especially the β1 subunit) is involved in achievement and retention of basolateral membrane residency in kidney cells [18]. Recently, kinase-mediated transporter regulation was revealed in the renal system. Renal tubular transport and reabsorption of water and electrolytes are regulated by two kinases, serine/threonine kinase Akt and serum-/glucocorticoid-inducible kinase 1 (SGK1), increasing Na^+^ reabsorption [19]. Signaling pathways involving the phosphorylation of Akt enhance activities of NBCe1, ENaC, and Na^+^-Cl^−^ cotransporter (NCC), and those involving SGK1 promote NHE3 activation, encouraging reabsorption of Na^+^. SGK1, furthermore, increases K^+^ secretion via the renal outer medullary potassium channel (ROMK) [19]. Major modulation of electrolyte transport occurs in the renal and exocrine systems, including salivary glands and pancreatic glands, and has already been extensively discussed [5,20]. In this review, we have focused on current knowledge of the regulation and roles of ion transporters in maintaining pH and appropriate HCO_3_^−^ concentration with respect to physiological and pathophysiological conditions of the various organs, except renal and exocrine systems. 

## 2. Carbonic Anhydrases: Regulation of HCO_3_^−^

In this section, we discuss the regulatory enzymes of HCO_3_^−^ transporters. The concentration of HCO_3_^−^ provides the driving force or favorable circumstances for transporters. As regulatory enzymes, various isoenzymes of carbonic anhydrase (CA) are differentially localized in the subcellular regions (cytosolic forms: CA I, CA II, CA III, CA VII and CA XIII; membrane-associated forms: CA IV, CA IX, CA XII, and CA XIV; mitochondria-associated forms: CA VA and CA VB; and secreted form: CA VI) and their catalytic activities vary. CAs participate in essential biological processes such as respiration, pH balance, and bicarbonate transport [21,22]. During the conversion of carbon dioxide to HCO_3_^−^, CAs are involved in HCO_3_^−^ movement, and participate in complexes of HCO_3_^−^ transporting systems [20]. Hydration of CO_2_ was considered an important source for HCO_3_^−^ [23,24]. Inhibition of CA with acetazolamide reduced duodenal HCO_3_^−^ secretion [23]. In addition, pH regulation of CA was addressed in various epithelia [25]. The role of CA on HCO_3_^−^ secretion was studied with HCO_3_^−^ transporters NBCn1 and NBCe2 in the duodenum [26]. Since CA was revealed to form a HCO_3_^−^ transport metabolon to accelerate HCO_3_^−^ flux, specific isoenzymes of CA have been implicated as the regulatory factor of HCO_3_^−^ transporters. CA II interacted with AE and DRA [27]. Our previous report addressed the fact that CA XII is physically associated with HCO_3_^−^ transporters anion exchanger 2 (AE2) and NBC, and regulates their activities [28]. The regulatory roles of CAs on the HCO_3_^−^ transporters have been reported to mediate cancer cell survival, cell migration, pH regulation, ion transport, and enamel formation in several biological systems [29,30,31,32,33,34,35,36]. Briefly, CA IX is involved in cell migration to facilitate the activities of AE2 and NBCe1 [30,31]. The role of oncogenic CAs will be discussed in Section 4.2. Inhibition of CA II and CA II knockout reduced the activity of NBCe1 in cortical astrocytes, suggesting that CA II is associated with an acid-loading role of NBCe1 into cells, and stabilized extracellular pH in brain tissue [33]. However, the precise regulation of CA isoforms and HCO_3_^−^ transporters remains unclear.

## 3. Regulatory Factors of HCO_3_^−^ Transporters

### 3.1. Regulatory Molecules

The HCO_3_^−^ transporters are mainly involved in the production of pancreatic and salivary fluid for HCO_3_^−^ secretion. The HCO_3_^−^-transporting mechanism is supported by several regulatory molecules and ions. This section focuses on the regulatory molecules of HCO_3_^−^ transporters, mostly found in secretory tissues such as salivary glands and pancreas [37,38,39,40]. The regulatory molecules include inositol-1,4,5-triphosphate (IP_3_) receptor binding protein released with IP_3_ (IRBIT), with-no-lysine (WNK) kinase, sterile 20 (STE20)-related proline/alanine-rich kinase (SPAK), spinophilin (SPL), and phosphatidylinositol 4,5-bisphosphate (PIP_2_) [20,41,42]. These factors mediate the supportive or inhibited function of HCO_3_^−^ transport via NBCe1-B, SLC26A6, and AE2 [20,41,42,43,44].

The IRBIT protein is abundantly expressed in various tissues. The IRBIT binds the NH_2_-terminal domain of IP_3_ receptors (IP_3_Rs) in resting state to inhibit the activity of IP_3_Rs; otherwise, the IRBIT is separated from IP_3_R when it stands in the stimulating state through the activation of G protein-coupled receptor [43,45]. The signaling cascade of released IRBIT is so far unknown. Mikoshiba et al. demonstrated that the IRBIT-binding site is located in the cytosolic N-terminus domain of NBCe-1B and clarified its interaction using a pull-down assay [46]. We previously reported that the N-terminus of NBCe1-B possesses a cluster of positively and negatively charged residues, and identified the positively charged cluster of the N-terminus of NBCe1-B as an IRBIT-binding domain [47]. The protein phosphatase 1 (PP1), as a supplementary protein of IRBIT, also possesses the enhanced role of NBCe1-B activity by the maintaining the membrane stability of NBCe1-B [38]. HCO_3_^−^ transporters are also regulated by WNK and SPAK kinases to suppress transportation of HCO_3_^−^ by decreasing the surface expression and the activity of HCO_3_^−^ transporters such as NBCe1-B and SLC26A6 [37,38]. WNKs mostly act with SPAK to phosphorylate and activate SPAK, and the activated SPAK subsequently phosphorylates NBCe1-B [38] and SLC26A6 [40,48]. The phosphorylation of these transporters decreases the membrane stability. The scaffolding functions of WNKs are mediated by their first 119 residues [49,50], and especially T-loops, to phosphorylate SPAK kinase [51]. SPAK kinases contain a proline- and arginine-rich domain (P/ARD), and a kinase domain including a serine motif (S-motif) and a COOH terminal (CCT) domain [51,52,53]. The CCT domain binds with SPAK binding motif [R/K]FX[V/I] of WNKs [54]. The SPAK phosphorylation site of NBCe1-B is S65 and T49 in the first 85 N-terminus residues, which acts as an autoinhibitory domain (AID) of NBCe1-B [39]. More recently, Lee et al. and Jeong et al. suggested that scaffolding protein SPL, as an actin cytoskeletal modulator, binds to AE2 and induces the enhanced AE2 activity [41,42]. SPL consists of a binding motif for F-actin, receptor, and canonical PP1 (R-K-I-H-F motif); PDZ domain; and three coiled-coil (C–C) domains [55]. Actually, the SPL can enhance AE2 activity through the 1–480 amino acid residues containing F-actin binding motif, receptor binding motif, and PP1 binding motif [42]. In addition, SPL activity is regulated by Ca^2+^ signaling with kinases including SPAK and Ca^2+^/calmodulin-dependent protein kinase II (CaMKII) [41]. PIP_2_ is the prevailing signaling molecule acting as a precursor of IP_3_ and stimulating NBCe1-A activity [56]. In addition, PIP_2_ indirectly increases NBCe1-B and NBCe1-C through increases of IP_3_ and Ca^2+^ [57]. The regulatory role of PIP_2_ on ion transporters can be considered a new therapeutic approach not only in NBCe1, but also in K_ATP_ [58,59], NHE1 [60], transient receptor potential canonical (TRPC) [61], and ATP-gated P2X channels [62,63]. Moreover, the activities of transporters are modulated by various phosphatase or kinase pathways and their multiple phosphorylation sites provide fine-tuning of transporters [64].

### 3.2. Cl^−^ as A Signaling Ion

The Concentration of Cl^−^ is responsible for regulating cellular functions to maintain fluid and electrolyte homeostasis through multiple transporters [65] such as CLC transporter [66], CBE [67], and CFTR [20,40]. The concentration of extracellular Cl^−^ controls the body fluid content of ions, cellular volume, and blood pressure [68]. Likewise, Cl^−^ plays a regulatory role in regulating HCO_3_^−^ absorption and secretion [69] and activity of NBC [70]. NBCe1-B in its resting state is inhibited by high concentrations of intracellular Cl^−^ ([Cl^−^]_i_) through the Cl^−^-interacting motif, GXXXP [70]. In NBCe1-B, the two GXXXP motifs sense intracellular Cl^−^, and one of the motifs is revealed by the autoinhibitory module of IRBIT which interacts with high affinity for Cl^−^. In resting state, the [Cl^−^]_i_ is maintained between 5 mM and 60 mM [71], which decreases activity of NBCe-1B to 40% of its highest activity, in contrast with a 140 mM concentration of Cl^−^, which inhibited 60% of NBCe-1B activity [70]. This complicated mechanism with differential affinity for Cl^−^ addresses that sensing with different range of [Cl^−^]_i_ saves NBCe1-B energy [70]. The [Cl^−^]_i_-sensing ability of GXXXP suggests a powerful regulatory factor; thus, further studies have to be conducted to control HCO_3_^−^ transportation more effectively.

## 4. HCO_3_^−^ and pH Regulation and Coordinated Transporters

Acid–base homeostasis with HCO_3_^−^ secretion is critically regulated in the kidney, lung, and exocrine systems such as salivary and pancreatic glands through various transporters, which have been extensively focused on in various reviews [5,20,72,73,74]. This section only focuses on pH regulation and the associated ion transporters including HCO_3_^−^ transporters in non-secretory systems. We have defined in this review that the non-secretory system includes the immune, tumorigenesis, tooth, vascular smooth muscle, heart, intestine, and reproduction systems.

### 4.1. Immune System

**Figure 1:**
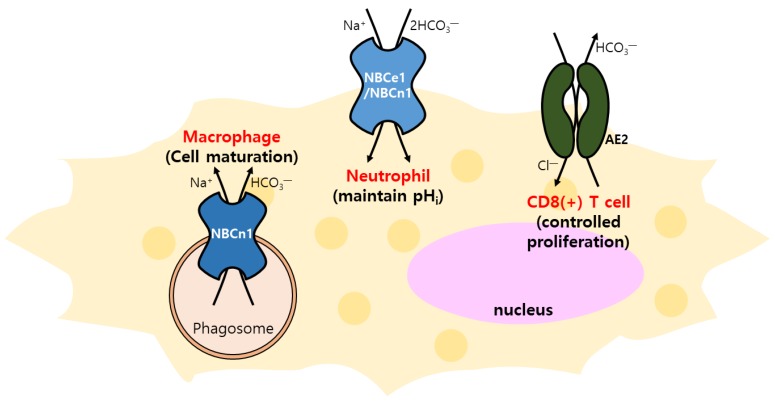
The regulation of immune cells through bicarbonate transporters. Immunological functions of NBC (NBCn1 and NBCe1) and AE2: macrophage maturation, maintenance pHi of neutrophil, and control of CD8^+^ T-cell proliferation.

HCO_3_^−^ transport is necessary for the immune defense system. For example, human lung cancer cells (Calu3) co-cultured with lymphocytes from CFTR-null mice showed abolished HCO_3_^−^ secretion in the host defense system due to bacterial infection [75]. Since NBC activity has been implicated in the pH regulation of lymphocytes, NBCn1 has recently been shown to be strongly induced upon macrophage differentiation to acidify phagosomes [76,77]. Loss of *SLC4A7* resulted in the increased intracellular acidification during phagocytosis, suggesting that intracellular pH homeostasis is associated with the anti-microbacterial function of macrophages [76]. Neutrophils also possess the HCO_3_^−^ transport mechanism involving NBCe1 or NBCn1, but not CBE, modulated by chemotactic agents, such as N-formylmethionyl-leucyl-phenylalanine (fMLF)/cytochalasin B, which regulates the basal pH_i_ [78,79]. Apart from the role of pH regulation, CD8^+^ T cells are dependent on AE2 as the CBE for modulation of cell proliferation and activation [80]. Primary biliary cirrhosis (PBC) is observed in progressive autoimmune-mediated cholangitis, and PBC patient reveal a reduced expression of AE2. The precise role of AE2 in PBC still remains unknown; however, AE2-deficient mice exhibit intrahepatic T-cell activation [81]. Currently, evidence of identified transporters and regulation of activity in the immune system is relatively rare compared to in other biological systems (Table 1 and Figure 1). The critical role of HCO_3_^−^ transport in the cellular network, including epithelial cells in immune cells, should be studied carefully in the future.

### 4.2. Tumor System

Modulation of pH is a primary process in cancer cells with high metabolic and proliferative features. The cancer cells undergo more acid-producing processes and produce acidic metabolites H^+^ and CO_2_. Therefore, adaptation of fluctuated extracellular pH is an essential process in tumors. Recently, a large cohort study on breast cancer, breast cancer cell lines, and a mouse model revealed enhanced expression of NHE1, NBCn1, and monocarboxylate transporters (MCT) MCT-1 and MCT-4 (Table 1) [82,83]. In addition, NBCe1 contributes to HCO_3_^−^ transport in the LS174 colon adenocarcinoma cell line and MDA-MB-231 breast cancer cells (Table 1) [31]. Disrupted NBCn1 expression delays murine breast cancer development and progression [83,84]. Tumorigenic signaling and induced tumor hypoxia in mal-perfused regions of tumors are main features of tumorigenesis. The expression of oncogenic CAs such as CA IX or CA XII is enhanced in hypoxic regions [85,86,87,88,89,90,91]. These enzymes help to maintain an acidic environment and produce HCO_3_^−^ to serve HCO_3_^−^ transporters. Several HCO_3_^−^ transporters interact with these CAs to facilitate the consumption of HCO_3_^−^ [28,35,92]. Although the role of CA in the tumorigenesis is beyond the scope of this section, HCO_3_^−^ transporters are, at least, upregulated and play critical roles in pH regulation in tumors [93] and will be further discussed in the final section. Data-mining analyses of changes in acid–base transporter expression revealed upregulated NHE1/3/4, various HCO_3_^−^ transporters (AE3 and DRA), H^+^ pumps, and MCT-4 in pancreatic ductal adenocarcinoma [94]. Following a different approach for hematological malignancies, treatment with HCO_3_^−^ transporter inhibitor 4-acetamido-4-isothio cyanostilbene-2,2-disulfonate (SITS) for T-cell lymphoma, designated Dalton’s lymphoma, increased the extracellular pH and induction of apoptosis [95]. Thus, blocking HCO_3_^−^ transport and disrupting pH homeostasis could be suggested as a promising anticancer therapy.

**Figure 2:**
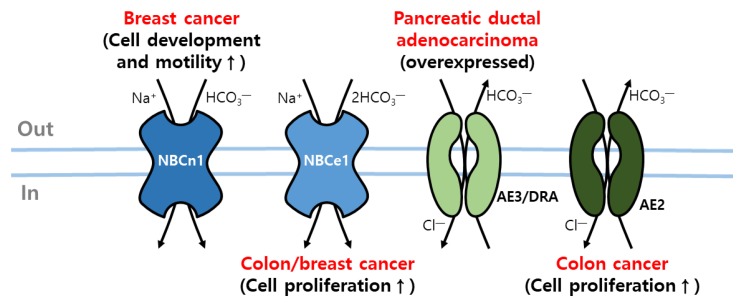
Upregulated bicarbonate transporters in cancer cells. The activation of NBCn1 and the expression of CBE (AE3 and DRA) is upregulated in breast cancer and pancreatic ductal adenocarcinoma. In addition, NBCe1 and AE2 activation increases colon cancer cell proliferation.

Beyond the role of pH regulation, several transporters are involved in cancer biology, including cell survival and migration (Table 1). Overexpression of AE2 in colon cancer is correlated with expression of Ki67 protein, a nuclear proliferation marker [96]. Enhanced expression of AE2 through the involvement of the transcription factor early growth response 1 (EGR1) promoted proliferation of colon cancer cells [96]. DRA is a critical Cl^−^/HCO_3_^−^ exchanger involved in absorption of Cl^−^ in the colon. The downregulation of a DRA gene (*SLC26A3*) is observed in adenomas and adenocarcinomas of the colon [97]. In addition to the proliferative colonic crypt zone, *SLC26A3*-null mice present high-Cl^−^-content diarrhea and more acidic lumen due to enhanced NHE3 expression and H^+^-ATPase as compensation of adaptive regulation [98]. The pH regulatory role of transporters in colon or intestinal units is discussed intensively in a later section. Moreover, NBCn1 expression is increased in human breast carcinoma tissue [99]. NHE1 and NBCn1 are involved in acid extrusion with different effects on the cathepsin release in breast cancer [100] and breast cancer cell motility [101]. They appear to serve as acid regulators (Figure 2). Although the role of ion transporters in cell migration is crucial in tissue homeostasis, this topic is not discussed in this review.

### 4.3. Tooth Developmental System

**Figure 3:**
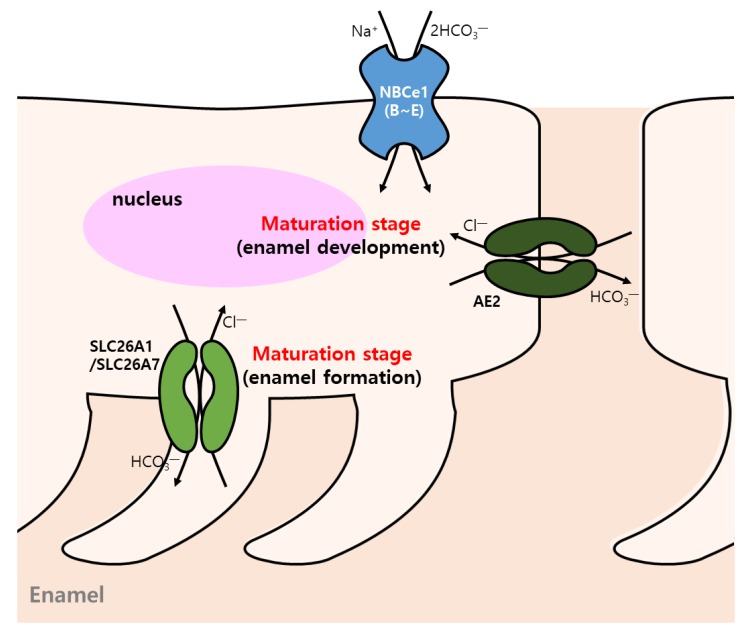
Enamel development induced by bicarbonate transporters. CBE (AE2, SLC26A1, and SLC26A7) and NBCs (NBCe1 (B–E)) transporters induce formation and development of enamel.

Enamel formation requires functional activity of ion transport for pH regulation. Ameloblasts in the maturation stage regulate extracellular pH to control the regulatory networks for enamel mineralization (Table 1) [102,103]. Modulation of pH fluctuation is mediated by the cyclic transformation of ruffle-ended ameloblasts and smooth-ended ameloblasts [103]. During amelogenesis, ameloblasts in maturation stage express the gene transcripts for NBCe1 isoforms B-E [104]. Mutation of NBCe1 is associated with defects of enamel development, and its expression in ameloblasts and papillary cells depends on the developmental process to modulate pH [104]. Matured ameloblasts secrete HCO_3_^−^ into the forming enamel through the involvement of AE2 [105]. In addition, ameloblasts express *SLC26A3, SLC26A4* (CBE), and *SLC26A6*, and the gene expression profile of null mice showed that *SLC26A* isoforms compensate their functions [105]. Moreover, SLC26A1 (CBE) and SLC26A7 (Cl^−^/base transporter) were also highly expressed in maturation-stage rodent ameloblasts [106] (Figure 3). *SLC26A1-* and *SLC26A7*-null mice did not exhibit abnormal enamel formation [106]. There were compensation processes of interaction units such as CFTR, CA II, CA VI, AE2, NBCe1, AE4, and SLC26A9 (epithelial Cl^−^ channel), and thus no obvious dental phenotype was revealed [102,106]. Bronckers summarized the major types of transmembrane molecules of ameloblasts [103]. The experimental and functional evidence of transporters and pH regulation in ameloblasts or dental cells needs to be elucidated. Recently, HCO_3_^−^ secretion was observed in ameloblasts from a rat cell line, HAT-7 cells, in a 2D culture system. HAT-7 cells are recommended as a useful functional model for transporters of ameloblasts [107]. Moreover, the characteristics of pH modulation in human dental pulp stem cells by NBC, NHE, AE, and Cl^−^/OH^−^ exchanger (CHE) were recently addressed [108].

### 4.4. Vascular Smooth Muscle System

Regulation of pH_i_ in vascular smooth muscle cells (VSMCs) has been studied in various disease models. Vascular wall affected by an agonist modulates pH to induce contraction or relaxation of mesenteric arteries. The regulation of NBC activity during artery contraction is associated with pH control (Table 1). The calcineurin A signaling pathway interacts with NBCn1, but not NHE, and modulates the NBC activity in VSMCs [109]. NBCn1-null mice were observed to exhibit attenuated myogenic tone in the presence of N-nitro-L-arginine methyl ester (L-NAME), and reduction in rhythmic contractile pattern compared to the wild type [110]. Disruption of NBCn1 inhibited nitric-oxide-mediated Rho kinase signaling and is involved in perturbed regulation of blood pressure [111]. The effect of alcohol on aortic smooth muscle cells modulated resting pH via the regulation of NBC, NHE, CHE, and AE [112]. Axial pH_i_ gradients enhanced migration and promoted filopodia via the NBCn1 in VSMCs, suggesting that NBCn1 and its HCO_3_^−^ component are involved in arterial remodeling [113,114]. Stimuli of NHE and NBCn1 under acidic extracellular pH inhibited NHE1 and NBCn1 activities in VSMCs [115].

### 4.5. Cardiac System

Acid–base balance is a critical factor in the cardiac system and is involved in excitation–contraction coupling. Thus, acid–base imbalance participates in cardiac dysfunction and arrhythmias. NHE1 in the gap junction at intercalated discs and NBC (not identified in subtype) in t-tubules were observed in rat ventricular myocytes to be involved in junction communication and excitation–contraction coupling, respectively [116].

The pathogenesis of cardiac tissue injury such as ischemic reperfusion injury involves various ion transporters such as NHE. During ischemia, the accumulation of intracellular H^+^ is mediated by NHE [117]. The Cl^−^/OX^2^^−^ exchanger/CBE SLC26A6 was detected and dominantly expressed in mouse cardiac myocytes to regulate pH balance [118,119]. However, the potential functions of cardiac SLC26A6 remain unknown. Recently, it has been reported that angiotensin II is responsible for the impairment of the NBCe1 in cardiac hypertrophy, and enhanced NBCn1 activity compensated the reduced function of NBCe1 (Table 1) [120]. NBCe1 is involved in myocardial damage by mediating Na^+^ and Ca^2+^ loading. Treatment with a-L3, a selective NBC inhibitor, improved myocardial function during ischemic reperfusion injury [120]. HCO_3_^−^ transporters functionally interact with carbonic anhydrases (CAs) [27,121,122,123]. The CA localized in the plasma membrane and cytosol and the CA-coupled NBC in plasma membrane modulated the intracellular pH to transport HCO_3_^−^ effectively into cytosol. Peetz et al. reported that the involvement of CA mediates the convergent NBC activity to modulate pH [35]. In addition, MCT activity enhanced lactate influx as an energy substrate in cardiomyocytes [35]. Although the modulatory mechanisms of NBC remain unclear, more recently, agonist stimulation of mineralocorticoid receptor, aldosterone, enhanced NBC activity via the activation of G-protein-coupled receptor GPR30/PI3K-AKT pathway in rat cardiomyocytes [124].

### 4.6. Digestive System

Duodenal, intestinal, and colonic HCO_3_^−^ regulation is crucial for epithelial defense against acid and for mucus secretion, as well as for pH regulation [125,126,127]. Several reports have shown that splice variants of NBC are differentially expressed in the intestinal system. NBCn1 is localized in duodenal villous enterocytes, colonic crypt cells, and goblet cells of the intestine [128]. Electrogenic splice variants of NBC, NBCe1-B and NBCe1-C, are predominantly expressed in proximal colon, and the electroneutral forms, NBCn1-C or NBCn1-D, are widely expressed in proximal and distal colon [129]. The parietal cells have been identified by AE2 in the basolateral membrane [130]. Recently, the Cl^−^/HCO_3_^−^ exchanger SLC26A9 has been reported in mouse and human gastrointestinal tract [131]. Reduced expression of SLC26A9 has also been related with impaired secretion of HCO_3_^−^ in the proximal duodenal mucus layer and enhanced death rate of CFTR-null mice [131]. The membrane expression of these transporters maintains intracellular pH homeostasis, and a potential role of coordinated CAs has been proposed in the gastrointestinal system [132]. Though the transporters are involved in HCO_3_^—^dependent mucosal function for epithelial protection of digestive tract (Table 1), the precise role and the differential expression of transporters and associated Cas should be studied more extensively.

### 4.7. Reproduction System

**Figure 4:**
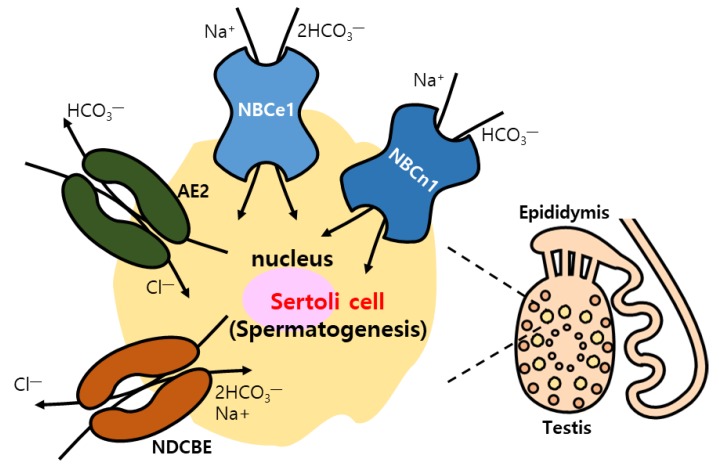
Male reproductive regulation with bicarbonate transporters. Spermatogenesis in sertoli cells induced by movement of bicarbonate ions through NBCe1, NBCn1, AE2, and NDCBE.

**Figure 5:**
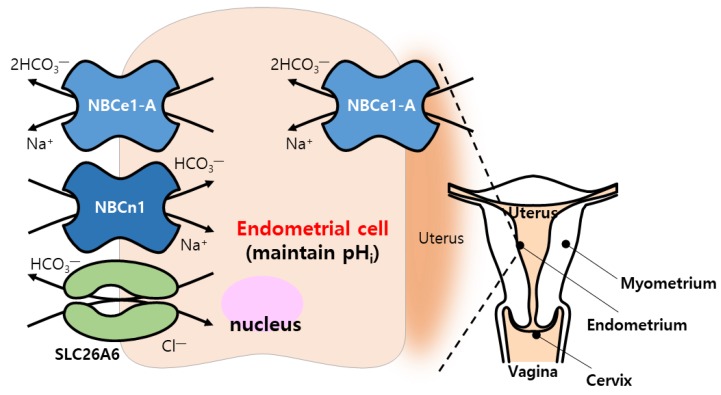
Female reproductive regulation with bicarbonate transporters. CBE (SLC26A6) and NBC (NBCn1 and NBCe1-A) transporters play an important role in maintenance of the pHi of endometrial cells.

HCO_3_^−^ is also critical to pH maintenance as well as ionic homeostasis in the reproduction system, including sperm production or sperm quality, male reproductive tract, and uterine epithelial cells [133]. AE2, NBCe1, NBCn1, and NDCBE were identified in sertoli cells, targets of hormonal signaling, and are associated with spermatogenesis [134,135] (Table 1 and Figure 4). Estradiol level is involved in the modulation of ion transporter expression [136,137,138,139,140]. CFTR also plays a critical role in male fertility. Age-dependent CFTR regulation correlates with sperm quality, including sperm motility. Reduced CFTR expression in sperm was observed in men with advanced age [141]. The male reproductive duct needs to maintain adequate luminal pH by modulating HCO₃^−^ transport through DRA, SLC26A6, and CFTR, and/or H^+^ transport through NHE3 [133,142,143,144]. In addition, SLC26A4 and SLC26A6 were expressed in the endometrial cells and localization of SLC26A6 was dependent on the menstrual cycle [145]. The uterine fluid is affected by changes in sex steroids due to the fluctuation in pH and Na^+^ concentration. Estradiol-induced NBCe1-A expression was enhanced in luminal and grandular epithelial cells of uteri [146]. Recently, NBCn1 present in the apical membrane of endometrial cells was also implicated in the balance between HCO_3_^−^ absorption and secretion [147] (Table 1 and Figure 5). However, in both males and females, the functional relationship between each transporter in maintenance of reproductive process needs to be elucidated more extensively.

**Table 1:** Identified HCO_3_^−^ transporters and their function in various system.

Physiological/Pathological System	Transporters	Localization and Function	References
Immune	NBCn1	Macrophage differentiation	[76,77]
	NBCe1, NBCn1	Neutrophils, maintenance of intracellular pH	[78,79]
	AE2	CD8^+^ T cells, controlling cell proliferation	[80]
Tumorigenesis	NBCe1	Colon/breast cancer, inducing cell proliferation	[31]
	NBCn1	Development and motility of breast cancer	[83,84,101]
	AE3, DRA	Pancreatic ductal adenocarcinoma	[94]
	AE2	Colon cancer, promotion of cell proliferation	[96]
Tooth development	NBCe1B–E, AE2	Ameloblasts in maturation stage, enamel development	[104,105]
	SLC26A1, SLC26A7	Maturation-stage rodent ameloblasts, enamel formation	[106]
	NBC, AE	Human dental pulp stem cells, pH modulation	[108]
Vascular smooth muscle	NBCn1	Vascular smooth muscle cells, myogenic tone, regulation of blood pressure, migration, arterial remodeling	[109], [110], [111], [112,113,114]
Cardiac	NBCe1, NBCn1	Cardiac hypertrophy	[120]
Digestive	NBCe1-B, NBCe1-C	Proximal colon	[129]
	NBCn1-C, NBCn1-D	Proximal and distal colon	[129]
	AE2	Parietal cells	[130]
	SLC26A9	GI tract	[131]
Reproduction	CFTR, AE2, NBCe1, NBCn1, NDCBE	Sertoli cells, spermatogenesis	[134,135]
	CFTR, DRA, SLC26A6	Male reproductive duct, maintenance of luminal pH	[133,142,143,144]
	NBCn1, SLC26A4, SLC26A6	Endometrial cells	[145,147]
	NBCe1-A	Luminal and grandular epithelial cells of uteri	[146]

## 5. Future Perspectives and Challenges

Various aspects of ion homeostasis and HCO_3_^−^ transporters in pH regulation and multiple cellular functions have been addressed in physiological and pathological systems. Throughout the body, impaired HCO_3_^−^ transport is associated with various diseases. However, the precise regulatory mechanisms are relatively unexplored and need to be identified for several systems. We summarized the expression and function of HCO_3_^−^ transporters and their associated CAs in various systems. Although all vital systems exhibit differential roles in maintaining their homeostasis, fundamental principles of transporter mechanism have emerged. Various splice variants of HCO_3_^−^ transporters exist. Moreover, the supportive role of CAs on HCO_3_^−^ transporters needs to be identified precisely. The combined modulation of ion transporters and CAs also needs to be elucidated and specified. In this study, understanding the correlation between these systems could be helpful in obtaining new insights into molecular HCO_3_^−^ signaling mechanisms and the potential therapeutic strategies.

## References

[1] Noor Z.N., Deitmer J.W., Theparambil S.M. (2018). Cytosolic sodium regulation in mouse cortical astrocytes and its dependence on potassium and bicarbonate. J. Cell. Physiol..

[2] Borowitz D. (2015). CFTR, bicarbonate, and the pathophysiology of cystic fibrosis. Pediatr. Pulmonol..

[3] Saint-Criq V., Gray M.A. (2017). Role of CFTR in epithelial physiology. Cell. Mol. Life Sci..

[4] Boron W.F., Fong P., Hediger M.A., Boulpaep E.L., Romero M.F. (1997). The electrogenic Na/HCO_3_ cotransporter. Kidney Int..

[5] Parker M.D., Boron W.F. (2013). The divergence, actions, roles, and relatives of sodium-coupled bicarbonate transporters. Physiol. Rev..

[6] Kopito R.R. (1990). Molecular biology of the anion exchanger gene family. Int. Rev. Cytol..

[7] Alper S.L., Sharma A.K. (2013). The *SLC26* gene family of anion transporters and channels. Mol. Asp. Med..

[8] Han Y., Shewan A.M., Thorn P. (2016). HCO_3_^−^ Transport through Anoctamin/Transmembrane Protein ANO1/TMEM16A in Pancreatic Acinar Cells Regulates Luminal pH. J. Biol. Chem..

[9] Sondo E., Caci E., Galietta L.J. (2014). The TMEM16A chloride channel as an alternative therapeutic target in cystic fibrosis. Int. J. Biochem. Cell Biol..

[10] Puga Molina L.C., Pinto N.A., Torres N.I., Gonzalez-Cota A.L., Luque G.M., Balestrini P.A., Romarowski A., Krapf D., Santi C.M., Trevino C.L. (2018). CFTR/ENaC-dependent regulation of membrane potential during human sperm capacitation is initiated by bicarbonate uptake through NBC. J. Biol. Chem..

[11] Collawn J.F., Matalon S. (2014). CFTR and lung homeostasis. Am. J. Physiol.-Lung Cell Mol. Physiol..

[12] Aalkjaer C., Boedtkjer E., Choi I., Lee S. (2014). Cation-coupled bicarbonate transporters. Compr. Physiol..

[13] Felder R.A., Jose P.A., Xu P., Gildea J.J. (2016). The Renal Sodium Bicarbonate Cotransporter NBCe2: Is It a Major Contributor to Sodium and pH Homeostasis?. Curr. Hypertens. Rep..

[14] Gildea J.J., Xu P., Kemp B.A., Carlson J.M., Tran H.T., Bigler Wang D., Langouet-Astrie C.J., McGrath H.E., Carey R.M., Jose P.A. (2018). Sodium bicarbonate cotransporter NBCe2 gene variants increase sodium and bicarbonate transport in human renal proximal tubule cells. PLoS ONE.

[15] Ng F.L., Boedtkjer E., Witkowska K., Ren M., Zhang R., Tucker A., Aalkjaer C., Caulfield M.J., Ye S. (2017). Increased NBCn1 expression, Na^+^/HCO_3_^−^ co-transport and intracellular pH in human vascular smooth muscle cells with a risk allele for hypertension. Hum. Mol. Genet..

[16] Pena-Munzenmayer G., George A.T., Shull G.E., Melvin J.E., Catalan M.A. (2016). Ae4 (Slc4a9) is an electroneutral monovalent cation-dependent Cl^−^/HCO_3_^−^ exchanger. J. Gen. Physiol..

[17] Kampik N.B., Gehring N., Schnitzbauer U., Hennings J.C., Hubner C.A., Wagner C.A. (2014). The murine Cl^−^/HCO_3_^−^ exchanger Ae3 (*Slc4a3*) is not required for acid-base balance but is involved in magnesium handling by the kidney. Cell. Physiol. Biochem..

[18] Su Y., Al-Lamki R.S., Blake-Palmer K.G., Best A., Golder Z.J., Zhou A., Karet Frankl F.E. (2015). Physical and functional links between anion exchanger-1 and sodium pump. J. Am. Soc. Nephrol..

[19] Satoh N., Nakamura M., Suzuki M., Suzuki A., Seki G., Horita S. (2015). Roles of Akt and SGK1 in the Regulation of Renal Tubular Transport. BioMed Res. Int..

[20] Lee M.G., Ohana E., Park H.W., Yang D., Muallem S. (2012). Molecular mechanism of pancreatic and salivary gland fluid and HCO3 secretion. Physiol. Rev..

[21] Şentürk M., Çavdar H., Talaz O., Supuran C.T. (2012). Carbonic Anhydrase Inhibitors and Activators: Small Organic Molecules as Drugs and Prodrugs. Medicinal Chemistry and Drug Design.

[22] Widdas W.F., Baker G.F., Baker P. (1994). The acceleration of pH volume changes in human red cells by bicarbonate and the role of carbonic anhydrase. Cytobios.

[23] Jacob P., Christiani S., Rossmann H., Lamprecht G., Vieillard-Baron D., Muller R., Gregor M., Seidler U. (2000). Role of Na^+^HCO_3_^−^ cotransporter NBC1, Na^+^/H^+^ exchanger NHE1, and carbonic anhydrase in rabbit duodenal bicarbonate secretion. Gastroenterology.

[24] Knutson T.W., Koss M.A., Hogan D.L., Isenberg J.I., Knutson L. (1995). Acetazolamide inhibits basal and stimulated HCO3- secretion in the human proximal duodenum. Gastroenterology.

[25] Mizumori M., Meyerowitz J., Takeuchi T., Lim S., Lee P., Supuran C.T., Guth P.H., Engel E., Kaunitz J.D., Akiba Y. (2006). Epithelial carbonic anhydrases facilitate PCO_2_ and pH regulation in rat duodenal mucosa. J. Physiol..

[26] Chen M., Praetorius J., Zheng W., Xiao F., Riederer B., Singh A.K., Stieger N., Wang J., Shull G.E., Aalkjaer C. (2012). The electroneutral Na^+^:HCO_3_^−^ cotransporter NBCn1 is a major pHi regulator in murine duodenum. J. Physiol..

[27] Sterling D., Brown N.J., Supuran C.T., Casey J.R. (2002). The functional and physical relationship between the DRA bicarbonate transporter and carbonic anhydrase II. Am. J. Physiol.-Cell Physiol..

[28] Hong J.H., Muhammad E., Zheng C., Hershkovitz E., Alkrinawi S., Loewenthal N., Parvari R., Muallem S. (2015). Essential role of carbonic anhydrase XII in secretory gland fluid and HCO_3_^−^ secretion revealed by disease causing human mutation. J. Physiol..

[29] Rafajova M., Zatovicova M., Kettmann R., Pastorek J., Pastorekova S. (2004). Induction by hypoxia combined with low glucose or low bicarbonate and high posttranslational stability upon reoxygenation contribute to carbonic anhydrase IX expression in cancer cells. Int. J. Oncol..

[30] Svastova E., Witarski W., Csaderova L., Kosik I., Skvarkova L., Hulikova A., Zatovicova M., Barathova M., Kopacek J., Pastorek J. (2012). Carbonic anhydrase IX interacts with bicarbonate transporters in lamellipodia and increases cell migration via its catalytic domain. J. Biol. Chem..

[31] Parks S.K., Pouyssegur J. (2015). The Na^+^/HCO_3_^−^ Co-Transporter SLC4A4 Plays a Role in Growth and Migration of Colon and Breast Cancer Cells. J. Cell. Physiol..

[32] Svichar N., Waheed A., Sly W.S., Hennings J.C., Hubner C.A., Chesler M. (2009). Carbonic anhydrases CA4 and CA14 both enhance AE3-mediated Cl^−^-HCO_3_^−^ exchange in hippocampal neurons. J. Neurosci..

[33] Theparambil S.M., Naoshin Z., Thyssen A., Deitmer J.W. (2015). Reversed electrogenic sodium bicarbonate cotransporter 1 is the major acid loader during recovery from cytosolic alkalosis in mouse cortical astrocytes. J. Physiol..

[34] Villafuerte F.C., Swietach P., Youm J.B., Ford K., Cardenas R., Supuran C.T., Cobden P.M., Rohling M., Vaughan-Jones R.D. (2014). Facilitation by intracellular carbonic anhydrase of Na^+^-HCO_3_^−^ co-transport but not Na^+^/H^+^ exchange activity in the mammalian ventricular myocyte. J. Physiol..

[35] Peetz J., Barros L.F., San Martin A., Becker H.M. (2015). Functional interaction between bicarbonate transporters and carbonic anhydrase modulates lactate uptake into mouse cardiomyocytes. Pflug. Arch.-Eur. J. Physiol..

[36] Lacruz R.S., Smith C.E., Moffatt P., Chang E.H., Bromage T.G., Bringas P., Nanci A., Baniwal S.K., Zabner J., Welsh M.J. (2012). Requirements for ion and solute transport, and pH regulation during enamel maturation. J. Cell. Physiol..

[37] Yang D., Shcheynikov N., Zeng W., Ohana E., So I., Ando H., Mizutani A., Mikoshiba K., Muallem S. (2009). IRBIT coordinates epithelial fluid and HCO_3_^−^ secretion by stimulating the transporters pNBC1 and CFTR in the murine pancreatic duct. J. Clin. Investig..

[38] Yang D., Li Q., So I., Huang C.L., Ando H., Mizutani A., Seki G., Mikoshiba K., Thomas P.J., Muallem S. (2011). IRBIT governs epithelial secretion in mice by antagonizing the WNK/SPAK kinase pathway. J. Clin. Investig..

[39] Hong J.H., Yang D., Shcheynikov N., Ohana E., Shin D.M., Muallem S. (2013). Convergence of IRBIT, phosphatidylinositol (4,5) bisphosphate, and WNK/SPAK kinases in regulation of the Na^+^-HCO_3_^−^ cotransporters family. Proc. Natl. Acad. Sci. USA.

[40] Park H.W., Nam J.H., Kim J.Y., Namkung W., Yoon J.S., Lee J.S., Kim K.S., Venglovecz V., Gray M.A., Kim K.H. (2010). Dynamic regulation of CFTR bicarbonate permeability by [Cl−]i and its role in pancreatic bicarbonate secretion. Gastroenterology.

[41] Lee D., Lee S.A., Shin D.M., Hong J.H. (2018). Chloride Influx of Anion Exchanger 2 Was Modulated by Calcium-Dependent Spinophilin in Submandibular Glands. Front. Physiol.

[42] Jeong Y.S., Hong J.H. (2016). Governing effect of regulatory proteins for Cl^−^/HCO_3_^−^ exchanger 2 activity. Channels.

[43] Hong J.H. (2015). Nanomaterials-Based Approaches for the Modulation of Sodium Bicarbonate Cotransporters. J. Nanomater..

[44] Thornell I.M., Bevensee M.O. (2015). Regulators of *Slc4* bicarbonate transporter activity. Front. Physiol..

[45] Ando H., Mizutani A., Kiefer H., Tsuzurugi D., Michikawa T., Mikoshiba K. (2006). IRBIT suppresses IP3 receptor activity by competing with IP3 for the common binding site on the IP3 receptor. Mol. Cell.

[46] Shirakabe K., Priori G., Yamada H., Ando H., Horita S., Fujita T., Fujimoto I., Mizutani A., Seki G., Mikoshiba K. (2006). IRBIT, an inositol 1,4,5-trisphosphate receptor-binding protein, specifically binds to and activates pancreas-type Na+/HCO3- cotransporter 1 (pNBC1). Proc. Natl. Acad. Sci. USA.

[47] Yang D.K., Shcheynikov N., Muallem S. (2011). IRBIT: It Is Everywhere. Neurochem. Res..

[48] Kahle K.T., Gimenez I., Hassan H., Wilson F.H., Wong R.D., Forbush B., Aronson P.S., Lifton R.P. (2004). WNK4 regulates apical and basolateral Cl^−^ flux in extrarenal epithelia. Proc. Natl. Acad. Sci. USA.

[49] He G., Wang H.R., Huang S.K., Huang C.L. (2007). Intersectin links WNK kinases to endocytosis of ROMK1. J. Clin. Investig..

[50] Heise C.J., Xu B.E., Deaton S.L., Cha S.K., Cheng C.J., Earnest S., Sengupta S., Juang Y.C., Stippec S., Xu Y.D. (2010). Serum and Glucocorticoid-induced Kinase (SGK) 1 and the Epithelial Sodium Channel Are Regulated by Multiple with No Lysine (WNK) Family Members. J. Biol. Chem..

[51] Richardson C., Alessi D.R. (2008). The regulation of salt transport and blood pressure by the WNK-SPAK/OSR1 signalling pathway. J. Cell Sci..

[52] Vitari A.C., Deak M., Morrice N.A., Alessi D.R. (2005). The WNK1 and WNK4 protein kinases that are mutated in Gordon’s hypertension syndrome phosphorylate and activate SPAK and OSR1 protein kinases. Biochem. J..

[53] Park S., Hong J.H., Ohana E., Muallem S. (2012). The WNK/SPAK and IRBIT/PP1 pathways in epithelial fluid and electrolyte transport. Physiology.

[54] Piechotta K., Garbarini N., England R., Delpire E. (2003). Characterization of the interaction of the stress kinase SPAK with the Na+-K+-2Cl^−^ cotransporter in the nervous system: Evidence for a scaffolding role of the kinase. J. Biol. Chem..

[55] Sarrouilhe D., di Tommaso A., Metaye T., Ladeveze V. (2006). Spinophilin: From partners to functions. Biochimie.

[56] Wu J.P., McNicholas C.M., Bevensee M.O. (2009). Phosphatidylinositol 4,5-bisphosphate (PIP2) stimulates the electrogenic Na/HCO_3_ cotransporter NBCe1-A expressed in Xenopus oocytes. Proc. Natl. Acad. Sci. USA.

[57] Thornell I.M., Wu J., Liu X., Bevensee M.O. (2012). PIP2 hydrolysis stimulates the electrogenic Na+-bicarbonate cotransporter NBCe1-B and -C variants expressed in Xenopus laevis oocytes. J. Physiol..

[58] Hilgemann D.W., Ball R. (1996). Regulation of cardiac Na^+^,Ca^2+^ exchange and K-ATP potassium channels by PIP2. Science.

[59] He Z.P., Feng S.Y., Tong Q.S., Hilgemann D.W., Philipson K.D. (2000). Interaction of PIP2 with the XIP region of the cardiac Na/Ca exchanger. Am. J. Physiol.-Cell Physiol..

[60] Aharonovitz O., Zaun H.C., Balla T., York J.D., Orlowski J., Grinstein S. (2000). Intracellular pH regulation by Na^+^/H^+^ exchange requires phosphatidylinositol 4,5-bisphosphate. J. Cell Biol..

[61] Soboloff J., Spassova M., Hewavitharana T., He L.P., Luncsford P., Xu W., Venkatachalam K., van Rossum D., Patterson R.L., Gill D.L. (2007). TRPC channels: Integrators of multiple cellular signals. Handb. Exp. Pharmacol..

[62] Bernier L.P., Ase A.R., Seguela P. (2013). Post-translational regulation of P2X receptor channels: Modulation by phospholipids. Front. Cell. Neurosci..

[63] Bernier L.P., Blais D., Boue-Grabot E., Seguela P. (2012). A dual polybasic motif determines phosphoinositide binding and regulation in the P2X channel family. PLoS ONE.

[64] Santos E., Crespo P. (2018). The RAS-ERK pathway: A route for couples. Sci. Signal..

[65] Luscher B.P., Vachel L., Ohana E., Muallem S. (2019). Cl^−^ as a bona fide signalling ion. Am. J. Physiol.-Cell Physiol..

[66] Dutzler R., Campbell E.B., Cadene M., Chait B.T., MacKinnon R. (2002). X-ray structure of a ClC chloride channel at 3.0 A reveals the molecular basis of anion selectivity. Nature.

[67] Yamaguchi M., Ishiguro H., Steward M., Sohma Y., Yamamoto A., Shimouchi A., Kondo T. (2009). Apical Cl^−^/HCO_3_^−^ exchanger stoichiometry in the modeling of HCO_3_^−^ transport by pancreatic duct epithelium. J. Med. Investig..

[68] Eladari D., Chambrey R., Picard N., Hadchouel J. (2014). Electroneutral absorption of NaCl by the aldosterone-sensitive distal nephron: Implication for normal electrolytes homeostasis and blood pressure regulation. Cell. Mol. Life Sci..

[69] Edwards A., Crambert G. (2017). Versatility of NaCl transport mechanisms in the cortical collecting duct. Am. J. Physiol.-Renal Physiol..

[70] Shcheynikov N., Son A., Hong J.H., Yamazaki O., Ohana E., Kurtz I., Shin D.M., Muallem S. (2015). Intracellular Cl^−^ as a signaling ion that potently regulates Na^+^/HCO_3_^−^ transporters. Proc. Natl. Acad. Sci. USA.

[71] Kahle K.T., Ring A.M., Lifton R.P. (2008). Molecular physiology of the WNK kinases. Annu. Rev. Physiol..

[72] Alka K., Casey J.R. (2014). Bicarbonate transport in health and disease. IUBMB Life.

[73] Levin L.R., Buck J. (2015). Physiological roles of acid-base sensors. Annu. Rev. Physiol..

[74] Atkinson K.F., Nauli S.M. (2016). pH sensors and ion Transporters: Potential therapeutic targets for acid-base disorders. Int. J. Pharma Res. Rev..

[75] Tang X.X., Fok K.L., Chen H., Chan K.S., Tsang L.L., Rowlands D.K., Zhang X.H., Dong J.D., Ruan Y.C., Jiang X. (2012). Lymphocyte CFTR promotes epithelial bicarbonate secretion for bacterial killing. J. Cell. Physiol..

[76] Sedlyarov V., Eichner R., Girardi E., Essletzbichler P., Goldmann U., Nunes-Hasler P., Srndic I., Moskovskich A., Heinz L.X., Kartnig F. (2018). The Bicarbonate Transporter SLC4A7 Plays a Key Role in Macrophage Phagosome Acidification. Cell Host Microbe.

[77] de la Rosa L.A., Cabado A.G., Botana M.A., Vieytes M.R., Vidal J.I., Botana L.M. (1999). Evidence for an electrogenic, negatively protein-kinase-A-modulated, Na^+^-dependent HCO_3_^−^ transporter in human lymphocytes. Pflug. Arch.-Eur. J. Physiol..

[78] Giambelluca M.S., Ciancio M.C., Orlowski A., Gende O.A., Pouliot M., Aiello E.A. (2014). Characterization of the Na/HCO3− cotransport in human neutrophils. Cell. Physiol. Biochem..

[79] Giambelluca M.S., Gende O.A. (2011). Cl^−^/HCO_3_^−^ exchange activity in fMLP-stimulated human neutrophils. Biochem. Biophys. Res. Commun..

[80] Concepcion A.R., Salas J.T., Sarvide S., Saez E., Ferrer A., Lopez M., Portu A., Banales J.M., Hervas-Stubbs S., Oude Elferink R.P. (2014). Anion exchanger 2 is critical for CD8(+) T cells to maintain pHi homeostasis and modulate immune responses. Eur. J. Immunol..

[81] Concepcion A.R., Salas J.T., Saez E., Sarvide S., Ferrer A., Portu A., Uriarte I., Hervas-Stubbs S., Oude Elferink R.P., Prieto J. (2015). CD8+ T cells undergo activation and programmed death-1 repression in the liver of aged Ae2a,b-/-mice favoring autoimmune cholangitis. Oncotarget.

[82] Andersen A.P., Samsoe-Petersen J., Oernbo E.K., Boedtkjer E., Moreira J.M.A., Kveiborg M., Pedersen S.F. (2018). The net acid extruders NHE1, NBCn1 and MCT4 promote mammary tumor growth through distinct but overlapping mechanisms. Int. J. Cancer.

[83] Lee S., Axelsen T.V., Andersen A.P., Vahl P., Pedersen S.F., Boedtkjer E. (2016). Disrupting Na^+^, HCO_3_^−^-cotransporter NBCn1 (*Slc4a7*) delays murine breast cancer development. Oncogene.

[84] Lee S., Mele M., Vahl P., Christiansen P.M., Jensen V.E., Boedtkjer E. (2015). Na^+^, HCO_3_^−^-cotransport is functionally upregulated during human breast carcinogenesis and required for the inverted pH gradient across the plasma membrane. Pflug. Arch.-Eur. J. Physiol..

[85] McIntyre A., Hulikova A., Ledaki I., Snell C., Singleton D., Steers G., Seden P., Jones D., Bridges E., Wigfield S. (2016). Disrupting Hypoxia-Induced Bicarbonate Transport Acidifies Tumor Cells and Suppresses Tumor Growth. Cancer Res..

[86] Wykoff C.C., Beasley N.J., Watson P.H., Turner K.J., Pastorek J., Sibtain A., Wilson G.D., Turley H., Talks K.L., Maxwell P.H. (2000). Hypoxia-inducible expression of tumor-associated carbonic anhydrases. Cancer Res..

[87] Watson P.H., Chia S.K., Wykoff C.C., Han C., Leek R.D., Sly W.S., Gatter K.C., Ratcliffe P., Harris A.L. (2003). Carbonic anhydrase XII is a marker of good prognosis in invasive breast carcinoma. Br. J. Cancer.

[88] Chiche J., Ilc K., Laferriere J., Trottier E., Dayan F., Mazure N.M., Brahimi-Horn M.C., Pouyssegur J. (2009). Hypoxia-inducible carbonic anhydrase IX and XII promote tumor cell growth by counteracting acidosis through the regulation of the intracellular pH. Cancer Res..

[89] Ambrosio M.R., Di Serio C., Danza G., Rocca B.J., Ginori A., Prudovsky I., Marchionni N., Del Vecchio M.T., Tarantini F. (2016). Carbonic anhydrase IX is a marker of hypoxia and correlates with higher Gleason scores and ISUP grading in prostate cancer. Diagn. Pathol..

[90] Finkelmeier F., Canli O., Peiffer K.H., Walter D., Tal A., Koch C., Pession U., Vermehren J., Trojan J., Zeuzem S. (2018). Circulating hypoxia marker carbonic anhydrase IX (CA9) in patients with hepatocellular carcinoma and patients with cirrhosis. PLoS ONE.

[91] Becker H.M. (2019). Carbonic anhydrase IX and acid transport in cancer. Br. J. Cancer.

[92] Becker H.M., Klier M., Deitmer J.W. (2014). Carbonic anhydrases and their interplay with acid/base-coupled membrane transporters. Subcell. Biochem..

[93] Gorbatenko A., Olesen C.W., Boedtkjer E., Pedersen S.F. (2014). Regulation and roles of bicarbonate transporters in cancer. Front. Physiol..

[94] Kong S.C., Giannuzzo A., Novak I., Pedersen S.F. (2014). Acid-base transport in pancreatic cancer: Molecular mechanisms and clinical potential. Biochem. Cell Biol..

[95] Kant S., Kumar A., Singh S.M. (2014). Bicarbonate transport inhibitor SITS modulates pH homeostasis triggering apoptosis of Dalton’s lymphoma: Implication of novel molecular mechanisms. Mol. Cell. Biochem..

[96] Song L.J., Liu R.J., Zeng Z., Alper S.L., Cui H.J., Lu Y., Zheng L., Yan Z.W., Fu G.H. (2012). Gastrin inhibits a novel, pathological colon cancer signaling pathway involving EGR1, AE2, and P-ERK. J. Mol. Med..

[97] Chapman J.M., Knoepp S.M., Byeon M.K., Henderson K.W., Schweinfest C.W. (2002). The colon anion transporter, down-regulated in adenoma, induces growth suppression that is abrogated by E1A. Cancer Res..

[98] Schweinfest C.W., Spyropoulos D.D., Henderson K.W., Kim J.H., Chapman J.M., Barone S., Worrell R.T., Wang Z., Soleimani M. (2006). *slc26a3* (dra)-deficient mice display chloride-losing diarrhea, enhanced colonic proliferation, and distinct up-regulation of ion transporters in the colon. J. Biol. Chem..

[99] Boedtkjer E., Moreira J.M., Mele M., Vahl P., Wielenga V.T., Christiansen P.M., Jensen V.E., Pedersen S.F., Aalkjaer C. (2013). Contribution of Na^+^, HCO_3_^−^-cotransport to cellular pH control in human breast cancer: A role for the breast cancer susceptibility locus NBCn1 (*SLC4A7*). Int. J. Cancer.

[100] Lauritzen G., Jensen M.B., Boedtkjer E., Dybboe R., Aalkjaer C., Nylandsted J., Pedersen S.F. (2010). NBCn1 and NHE1 expression and activity in DeltaNErbB2 receptor-expressing MCF-7 breast cancer cells: Contributions to pHi regulation and chemotherapy resistance. Exp. Cell Res..

[101] Lauritzen G., Stock C.M., Lemaire J., Lund S.F., Jensen M.F., Damsgaard B., Petersen K.S., Wiwel M., Ronnov-Jessen L., Schwab A. (2012). The Na^+^/H^+^ exchanger NHE1, but not the Na^+^, HCO_3_^−^ cotransporter NBCn1, regulates motility of MCF7 breast cancer cells expressing constitutively active ErbB2. Cancer Lett..

[102] Yin K., Paine M.L. (2017). Bicarbonate Transport During Enamel Maturation. Calcif. Tissue Int..

[103] Bronckers A.L. (2017). Ion Transport by Ameloblasts during Amelogenesis. J. Dent. Res..

[104] Jalali R., Guo J., Zandieh-Doulabi B., Bervoets T.J., Paine M.L., Boron W.F., Parker M.D., Bijvelds M.J., Medina J.F., DenBesten P.K. (2014). NBCe1 (*SLC4A4*) a potential pH regulator in enamel organ cells during enamel development in the mouse. Cell Tissue Res..

[105] Jalali R., Zandieh-Doulabi B., DenBesten P.K., Seidler U., Riederer B., Wedenoja S., Micha D., Bronckers A.L. (2015). *Slc26a3*/Dra and *Slc26a6* in Murine Ameloblasts. J. Dent. Res..

[106] Yin K., Lei Y., Wen X., Lacruz R.S., Soleimani M., Kurtz I., Snead M.L., White S.N., Paine M.L. (2015). *SLC26A* Gene Family Participate in pH Regulation during Enamel Maturation. PLoS ONE.

[107] Bori E., Guo J., Racz R., Burghardt B., Foldes A., Keremi B., Harada H., Steward M.C., Den Besten P., Bronckers A.L. (2016). Evidence for Bicarbonate Secretion by Ameloblasts in a Novel Cellular Model. J. Dent. Res..

[108] Chen G.S., Lee S.P., Huang S.F., Chao S.C., Chang C.Y., Wu G.J., Li C.H., Loh S.H. (2018). Functional and molecular characterization of transmembrane intracellular pH regulators in human dental pulp stem cells. Arch. Oral Biol..

[109] Danielsen A.A., Parker M.D., Lee S., Boron W.F., Aalkjaer C., Boedtkjer E. (2013). Splice cassette II of Na^+^, HCO_3_^−^ cotransporter NBCn1 (*slc4a7*) interacts with calcineurin A: Implications for transporter activity and intracellular pH control during rat artery contractions. J. Biol. Chem..

[110] Thomsen A.B., Kim S., Aalbaek F., Aalkjaer C., Boedtkjer E. (2014). Intracellular acidification alters myogenic responsiveness and vasomotion of mouse middle cerebral arteries. J. Cereb. Blood Flow Metab..

[111] Boedtkjer E., Praetorius J., Matchkov V.V., Stankevicius E., Mogensen S., Fuchtbauer A.C., Simonsen U., Fuchtbauer E.M., Aalkjaer C. (2011). Disruption of Na^+^, HCO_3_^−^ cotransporter NBCn1 (*slc4a7*) inhibits NO-mediated vasorelaxation, smooth muscle Ca^2+^ sensitivity, and hypertension development in mice. Circulation.

[112] Loh S.H., Lee C.Y., Chen G.S., Wu C.H., Tsao C.J., Shih S.J., Chou C.C., Tsai C.S., Tsai Y.T. (2015). The Effect and Underlying Mechanism of Ethanol on Intracellular H^+^-Equivalent Membrane Transporters in Human Aorta Smooth Muscle Cells. Alcohol. Clin. Exp. Res..

[113] Boedtkjer E., Bentzon J.F., Dam V.S., Aalkjaer C. (2016). Na^+^, HCO_3_^−^-cotransporter NBCn1 increases pH gradients, filopodia, and migration of smooth muscle cells and promotes arterial remodelling. Cardiovasc. Res..

[114] Boedtkjer E., Praetorius J., Aalkjaer C. (2006). NBCn1 (*slc4a7*) mediates the Na+-dependent bicarbonate transport important for regulation of intracellular pH in mouse vascular smooth muscle cells. Circ. Res..

[115] Bonde L., Boedtkjer E. (2017). Extracellular acidosis and very low [Na(+) ] inhibit NBCn1- and NHE1-mediated net acid extrusion from mouse vascular smooth muscle cells. Acta Physiol..

[116] Garciarena C.D., Ma Y.L., Swietach P., Huc L., Vaughan-Jones R.D. (2013). Sarcolemmal localisation of Na^+^/H^+^ exchange and Na^+^-HCO_3_^−^ co-transport influences the spatial regulation of intracellular pH in rat ventricular myocytes. J. Physiol..

[117] Karmazyn M., Moffat M.P. (1993). Role of Na^+^/H^+^ exchange in cardiac physiology and pathophysiology: Mediation of myocardial reperfusion injury by the pH paradox. Cardiovasc. Res..

[118] Alvarez B.V., Kieller D.M., Quon A.L., Markovich D., Casey J.R. (2004). Slc26a6: A cardiac chloride-hydroxyl exchanger and predominant chloride-bicarbonate exchanger of the mouse heart. J. Physiol..

[119] Kim H.J., Myers R., Sihn C.R., Rafizadeh S., Zhang X.D. (2013). Slc26a6 functions as an electrogenic Cl^−^/HCO_3_^−^ exchanger in cardiac myocytes. Cardiovasc. Res..

[120] Fantinelli J.C., Orlowski A., Aiello E.A., Mosca S.M. (2014). The electrogenic cardiac sodium bicarbonate co-transporter (NBCe1) contributes to the reperfusion injury. Cardiovasc. Pathol..

[121] Alvarez B.V., Loiselle F.B., Supuran C.T., Schwartz G.J., Casey J.R. (2003). Direct extracellular interaction between carbonic anhydrase IV and the human NBC1 sodium/bicarbonate co-transporter. Biochemistry.

[122] Casey J.R., Sly W.S., Shah G.N., Alvarez B.V. (2009). Bicarbonate homeostasis in excitable tissues: Role of AE3 Cl^−^/HCO_3_^−^ exchanger and carbonic anhydrase XIV interaction. Am. J. Physiol.-Cell Physiol..

[123] Morgan P.E., Pastorekova S., Stuart-Tilley A.K., Alper S.L., Casey J.R. (2007). Interactions of transmembrane carbonic anhydrase, CAIX, with bicarbonate transporters. Am. J. Physiol.-Cell Physiol..

[124] De Giusti V.C., Orlowski A., Ciancio M.C., Espejo M.S., Gonano L.A., Caldiz C.I., Vila Petroff M.G., Villa-Abrille M.C., Aiello E.A. (2015). Aldosterone stimulates the cardiac sodium/bicarbonate cotransporter via activation of the g protein-coupled receptor gpr30. J. Mol. Cell. Cardiol..

[125] Safsten B. (1993). Duodenal bicarbonate secretion and mucosal protection. Neurohumoral influence and transport mechanisms. Acta Physiol. Scand. Suppl..

[126] Rossmann H., Bachmann O., Vieillard-Baron D., Gregor M., Seidler U. (1999). Na^+^/HCO_3_^−^ cotransport and expression of NBC1 and NBC2 in rabbit gastric parietal and mucous cells. Gastroenterology.

[127] Seidler U., Rossmann H., Jacob P., Bachmann O., Christiani S., Lamprecht G., Gregor M. (2000). Expression and function of Na+HCO3− cotransporters in the gastrointestinal tract. Ann. N. Y. Acad. Sci..

[128] Singh A.K., Xia W., Riederer B., Juric M., Li J., Zheng W., Cinar A., Xiao F., Bachmann O., Song P. (2013). Essential role of the electroneutral Na^+^-HCO_3_^−^ cotransporter NBCn1 in murine duodenal acid-base balance and colonic mucus layer build-up in vivo. J. Physiol..

[129] Barmeyer C., Ye J.H., Soroka C., Geibel P., Hingsammer L.M., Weitgasser L., Atway D., Geibel J.P., Binder H.J., Rajendran V.M. (2013). Identification of functionally distinct Na-HCO3 co-transporters in colon. PLoS ONE.

[130] Stuart-Tilley A., Sardet C., Pouyssegur J., Schwartz M.A., Brown D., Alper S.L. (1994). Immunolocalization of anion exchanger AE2 and cation exchanger NHE-1 in distinct adjacent cells of gastric mucosa. Am. J. Physiol..

[131] Liu X., Li T., Riederer B., Lenzen H., Ludolph L., Yeruva S., Tuo B., Soleimani M., Seidler U. (2015). Loss of *Slc26a9* anion transporter alters intestinal electrolyte and HCO_3_^−^ transport and reduces survival in CFTR-deficient mice. Pflug. Arch. Eur. J. Physiol..

[132] Niv Y., Fraser G.M. (2002). The alkaline tide phenomenon. J. Clin. Gastroenterol..

[133] Chan H.C., Sun X. (2014). SLC26 anion exchangers in uterine epithelial cells and spermatozoa: Clues from the past and hints to the future. Cell Biol. Int..

[134] Bernardino R.L., Martins A.D., Jesus T.T., Sa R., Sousa M., Alves M.G., Oliveira P.F. (2015). Estrogenic regulation of bicarbonate transporters from SLC4 family in rat Sertoli cells. Mol. Cell. Biochem..

[135] Bernardino R.L., Costa A.R., Martins A.D., Silva J., Barros A., Sousa M., Sa R., Alves M.G., Oliveira P.F. (2016). Estradiol modulates Na^+^ -dependent HCO_3_^−^ transporters altering intracellular pH and ion transport in human Sertoli cells: A role on male fertility?. Biol. Cell.

[136] Bi R.Y., Meng Z., Zhang P., Wang X.D., Ding Y., Gan Y.H. (2017). Estradiol upregulates voltage-gated sodium channel 1.7 in trigeminal ganglion contributing to hyperalgesia of inflamed TMJ. PLoS ONE.

[137] Ren P., Wang W.B., Pan H.H., Qiu C.Y., Hu W.P. (2018). Up-regulation of ASIC3 expression by beta-estradiol. Neurosci. Lett..

[138] Yang X., Mao X., Xu G., Xing S., Chattopadhyay A., Jin S., Salama G. (2018). Estradiol up-regulates L-type Ca^2+^ channels via membrane-bound estrogen receptor/phosphoinositide-3-kinase/Akt/cAMP response element-binding protein signaling pathway. Heart Rhythm.

[139] Hill B.J.F., Dalton R.J., Joseph B.K., Thakali K.M., Rusch N.J. (2017). 17beta-estradiol reduces Cav 1.2 channel abundance and attenuates Ca^2+^-dependent contractions in coronary arteries. Pharmacol. Res. Perspect..

[140] Luo L., Deng J., Wang D.X., He J., Deng W. (2015). Regulation of epithelial sodium channel expression by oestradiol and progestogen in alveolar epithelial cells. Respir. Physiol. Neurobiol..

[141] Diao R., Fok K.L., Zhao L., Chen H., Tang H., Chen J., Zheng A., Zhang X., Gui Y., Chan H.C. (2013). Decreased expression of cystic fibrosis transmembrane conductance regulator impairs sperm quality in aged men. Reproduction.

[142] Pierucci-Alves F., Akoyev V., Stewart J.C., Wang L.H., Janardhan K.S., Schultz B.D. (2011). Swine models of cystic fibrosis reveal male reproductive tract phenotype at birth. Biol. Reprod..

[143] Hihnala S., Kujala M., Toppari J., Kere J., Holmberg C., Hoglund P. (2006). Expression of SLC26A3, CFTR and NHE3 in the human male reproductive tract: Role in male subfertility caused by congenital chloride diarrhoea. Mol. Hum. Reprod..

[144] Zhou Q., Clarke L., Nie R., Carnes K., Lai L.W., Lien Y.H., Verkman A., Lubahn D., Fisher J.S., Katzenellenbogen B.S. (2001). Estrogen action and male fertility: Roles of the sodium/hydrogen exchanger-3 and fluid reabsorption in reproductive tract function. Proc. Natl. Acad. Sci. USA.

[145] Suzuki K., Royaux I.E., Everett L.A., Mori-Aoki A., Suzuki S., Nakamura K., Sakai T., Katoh R., Toda S., Green E.D. (2002). Expression of PDS/Pds, the Pendred syndrome gene, in endometrium. J. Clin. Endocrinol. Metab..

[146] Gholami K., Muniandy S., Salleh N. (2014). Modulation of sodium-bicarbonate co-transporter (*SLC4A4*/NBCe1) protein and mRNA expression in rat’s uteri by sex-steroids and at different phases of the oestrous cycle. Res. Vet. Sci..

[147] Xie Z.D., Guo Y.M., Ren M.J., Yang J., Wang S.F., Xu T.H., Chen L.M., Liu Y. (2018). The Balance of HCO_3_^−^ Secretion vs. Reabsorption in the Endometrial Epithelium Regulates Uterine Fluid pH. Front. Physiol..

